# Effectiveness of Alternative Methods for Toothbrush Disinfection: An *In Vitro* Study

**DOI:** 10.1155/2014/726190

**Published:** 2014-05-25

**Authors:** Ilkay Peker, Gulcin Akca, Cigdem Sarikir, Meryem Toraman Alkurt, Irem Celik

**Affiliations:** ^1^Department of Dentomaxillofacial Radiology, Gazi University Faculty of Dentistry, 82 Sokak, No. 4, Emek, 06510 Ankara, Turkey; ^2^Department of Medical Microbiology, Gazi University Faculty of Dentistry, 82 Sokak, No. 4, Emek, 06510 Ankara, Turkey

## Abstract

*Objective.* This study aimed to evaluate the effectiveness of alternative methods for toothbrush disinfection. *Methods.* Two-hundred eighty toothbrushes were included in the study. The toothbrushes were divided into 7 groups and were contaminated by standardized suspensions of *Lactobacillus rhamnosus (L. rhamnosus), Streptococcus mutans (S. mutans), Staphylococcus aureus (S. aureus),* and *Escherichia coli (E. coli*). The following disinfectants were tested: 1% sodium hypochlorite (NaOCl), 100% and 50% white vinegar, microwave (MW) oven, ultraviolet (UV) sanitizer, and mouth rinse-containing propolis (MCP). Data were analyzed with Kruskal Wallis and Dunn's tests. *Results.* Statistically significant differences were found between different methods and control group for all tested bacteria. There were statistically significant differences between all test groups for all microorganisms. MW was the most effective for *L. rhamnosus* and 100% white vinegar was the most effective method for *S. mutans* and *S. aureus*. NaOCl was the most effective for *E. coli*. *Conclusion.* This study showed that 100% white vinegar was considered to be effective for tested microorganisms. Similarly, 1% NaOCl is cost-effective, easily accessible, and comparatively effective for toothbrush disinfection. Because these agents are nontoxic, cost-effective and easily accessible, they may be appropriate for household use.

## 1. Introduction

Toothbrushes are necessary for daily oral hygiene, but residues remaining on their bristles may precipitate the growth of several microorganisms [1]. Over 700 bacterial species, as well as fungi, viruses, and transient microorganisms, are present in the oral cavity that may or may not cause various diseases [2].

As early as 1920, Cobb reported the toothbrush as a cause of repeated infections in the mouth [3]. Many bacteria are found in toothbrushes after brushing [4], and the microorganisms maintain their viability, ranging from one day to one week [5]. In addition, toothbrushes are frequently stored in the bathroom or close to the toilet and sink and may be exposed to enteric bacteria dispersed by aerosols [6]. Even small droplets from the toilet lead to the release of millions of bacteria into the atmosphere [7]. The contamination mostly increases when toothbrushes are shared or stored together. Several factors, including the long survival time of the microorganisms, storage circumstances, and toothbrush location, cause the reintroduction of potential pathogens and cross-infection to the oral cavity [7].

Contaminated toothbrushes may play an important role in many oral and systemic diseases, including septicemia and gastrointestinal, cardiovascular, respiratory, and renal problems [8]. Some studies have suggested the need for disinfecting toothbrushes to prevent various diseases using different methods [9, 10]. This condition is specifically important for children, the elderly, and high-risk patients, including immunosuppressed individuals or those undergoing organ transplantation or chemotherapy [8].

Although different methods have been investigated for toothbrush disinfection in the literature, this matter has received little attention by many researchers because most clinicians still consider toothbrushes only as caries and plaque controlling devices [7].

This study aimed to evaluate the effectiveness of 1% sodium hypochlorite (NaOCl) solution, microwave (MW) oven, UV (UV) sanitizer, mouth rinse-containing propolis (MCP), 50% white vinegar, and 100% white vinegar for toothbrush disinfection.

## 2. Methods

*Staphylococcus aureus (S. aureus) ATCC#25923, Streptococcus mutans (S. mutans) ATCC#25175, Escherichia coli (E. coli) ATCC#25922,* and* Lactobacillus rhamnosus (L. rhamnosus) ATCC#9595* strains were used in the study.* S. aureus* and* E. coli* were cultured in brain heart infusion broth (BHI, Merck, Germany) at 37°C for 24 hours aerobically.* S. mutans* was cultured in trypticase soy broth (TYC) (Merck, Germany).* L. rhamnosus* was cultured in MRS broth (Merck, Germany) at 37°C for 48–72 hours with an atmosphere of 5% CO_2_. After incubation, the bacterial inoculum was adjusted to 1.5 ×10^8^ colony forming units/mL (cfu/mL) according to the 0.5 McFarland test standard.

Two-hundred eighty toothbrushes with standardized dimensions, bristles, and trademarks (Colgate Palmolive Corp., Istanbul, Turkey) were included, and 6 different methods were used in the study. After autoclave sterilization, the toothbrushes were divided into 7 groups (6 test groups and 1 control group, *n* = 10) according to the microorganisms and the method (Table 1).

Standardized suspensions of the chosen microorganisms were adjusted to 1 × 10^6^ colony forming unit per mL (cfu/mL) at a density and confirmed by measuring their optical density (OD) spectrophotometrically using an ELISA reader (OD: 0.600–0.625) (Biotek ELx800, USA). Each toothbrush was immersed into test tubes including 10 mL of BHI for* S. aureus* and* E. coli*, TYC for* S. mutans* and MRS for* L. rhamnosus*. Then, they were infected with 100 *μ*L of each bacterial inoculum as previously mentioned. The* S. mutans*- and* L. rhamnosus*-infected toothbrushes were placed in an incubator at 37°C with 5% CO_2_ for 24–48 hours, and the* S. aureus*- and* E. coli*-infected test tubes were placed in an aerobic incubator at 37°C for 24 hours. After incubation, the toothbrushes were removed from the tubes gently and washed three times with 5 mL of phosphate-buffered saline solution (PBS) (pH 7.2). Then, they were individually placed in test tubes including 10 mL of 1% NaOCl, 50% or 100% white vinegar in sterilized deionized water, or ready-to-use MCP (Fresh-up Cosmetics, Varna, Bulgaria) for 10 min. Then, the toothbrushes were removed from the tubes, placed in other sterilized test tubes with distilled water, washed 3 times gently, and placed in other test tubes with 10 mL of sterilized distilled water. In another group, the toothbrushes were placed in a UV sanitizer (Dental Total Status) for 20 minutes for both sides of the brushes. In another group, the toothbrushes were placed in a microwave oven at 650 watts for 3 minutes for both sides of the brushes. Then, the toothbrushes were placed in test tubes with 10 mL of sterilized distilled water. For the control group, the infected toothbrushes were placed in 10 mL of sterilized distilled water. After vortexing rigorously for 1 minute, all of the tubes were diluted to 10^−2^ and 10^−3^, and 25 *μ*L aliquots of the specimens were seeded onto BHI agar (Merck, Germany) for* S. aureus *and* E. coli*, onto TYC agar (Merck, Germany) for* S. mutans,* and onto MRS agar (Merck, Germany) for* L. rhamnosus*. After incubating as mentioned before, the grown colonies were counted, and the number of colonies was calculated according to the dilution ratio and defined as cfu/mL.

### 2.1. Data Analysis

The mean values, standard deviations, and medians for the data for each microorganism after disinfection with the different methods were calculated with descriptive statistics. The data were analyzed using Kruskal Wallis and Dunn's nonparametrical tests with 95% confidence intervals for multiple comparisons.

## 3. Results

Statistically significant differences (*P* < 0.05) were found between the different methods (Groups 1, 2, 3, 4, 5, and 6) and control group (Group 7) for all of the tested bacteria. There were statistically significant differences (*P* < 0.05) between the groups (Groups 1, 2, 3, 4, 5, and 6) of all of the tested methods for all of the microorganisms. MW was the most effective for* L. rhamnosus* followed by 100% white vinegar, 1% NaOCl, 50% white vinegar, UV, and MCP (Table 2). Additionally, 100% white vinegar was found to be the most effective method for* S. mutans* followed by 1% NaOCl, 50% white vinegar, UV, MW, and MCP (Table 3). Furthermore, 100% white vinegar was found to be the most effective for* S. aureus* followed by 1% NaOCl, MW, UV, 50% white vinegar, and MCP (Table 4). For* E. coli*, 1% NaOCl was the most effective followed by 100% white vinegar, 50% white vinegar, MW, UV, and MCP (Table 5). Although statistically significant differences (*P* < 0.05) were found between MCP and the control group, this method was found to be less effective for toothbrush disinfection.

## 4. Discussion

Previous studies have reported that toothbrushes become contaminated by several oral microorganisms after oral hygiene procedures, and contaminated toothbrushes may transmit bacteria involved in oral and systemic diseases [7, 9, 11]. Nevertheless, toothbrush disinfection has not been considered by oral health professionals, and the subject has not received enough attention in the literature. In addition, there are very few products for this purpose on the market.

The efficacy of different methods for disinfecting toothbrushes has been investigated in* in vitro* and* in vivo* studies. Methods such as chemical agents, brush sprays, UV light toothbrush sanitizers, modified brushes, MW ovens, and dishwashers have been suggested for disinfecting toothbrushes [7, 9, 12, 13].

Oral bacteria with pathogenic potential, including* S. mutans*,* L. rhamnosus*,* S. aureus*, and* E. coli*, were selected for this study.* S. mutans*, known as major bacteria related to dental caries, is considered to be one of the causative agents for infectious endocarditis, especially in children with congenital heart disease (IE) [13, 14].* L. rhamnosus *is characteristically known to lead to the progression of carious lesions, and bacteremia, meningitis, and endocarditis have been reported, particularly in immunocompromised patients [15].* S. aureus* causes several diseases, such as pneumonia, sepsis, abscesses, infective endocarditis, and osteomyelitis [16].* E. coli* is an important cause of diarrhea, urinary tract infections, and septicemia [17]. Several microorganisms, including* E. coli*, have been found on toothbrushes kept in the bathroom for 3 months [17]. The efficacy of different toothbrush disinfection methods for* S. mutans*,* S. aureus*, and* E. coli* was investigated in previous studies [12, 18]. According to our knowledge, the influence of the toothbrush disinfection method for* L. rhamnosus* has not been studied. In this study, the toothbrushes were contaminated with* S. mutans*,* L. rhamnosus*,* S. aureus*, and* E. coli*.

NaOCl is widely used as the main root canal irrigant because of its broad antimicrobial activity in endodontics. Although the cytotoxic effect of this agent has been reported at different concentrations for vital tissues [19], the cytotoxic properties of 2–2.5% NaOCl do not appear during short-term exposure, and no genotoxic effect has been found for host tissues [20]. Mobin et al. investigated fungal contamination in toothbrushes and suggested that 2% NaOCl is effective for 3–5 minutes and is a low-cost method to disinfect a toothbrush [1]. Da Silva et al. investigated the effectiveness of different disinfectant solutions for disinfecting acrylic resin specimens contaminated with* Candida albicans* (*C. albicans*),* S. mutans*,* S. aureus*,* E. coli*, and* Bacillus subtilis* [21]. They found 1% NaOCl to be the best antimicrobial agent against the tested microorganisms [21]. This result is supported by Salvia et al. [22]. Similarly, in this study, 1% NaOCl significantly reduced the counts for all of the tested microorganisms, eradicated virtually all of* S. mutans*, and was found to be the most effective method for* E. coli*. This result is in accordance with previous studies.

MW irradiation is an effective method to sterilize acrylic resins [23]. The antimicrobial effect of MW irradiation has been shown for removable dentures contaminated with* Streptococcus epidermidis*,* S. aureus*,* Klebsiella pneumonia*,* Bacillus subtilis*, and* C. albicans* for 6–10 minutes [23]. There are a few studies on the efficiency of MW irradiation for toothbrushes [12, 18]. Bélanger-Giguére et al. studied MW irradiation on high power for 5 minutes to disinfect toothbrushes contaminated with* S. mutans*, and they reported that the method was effective but could not completely eradicate the microorganisms. In addition, the toothbrushes became unusable after irradiation for 5 minutes [18]. Spolidorio et al. applied MW irradiation at 650 watts for 1 minute to disinfect toothbrushes and tongue scrapers contaminated with* C. albicans*,* S. mutans*, and* S. aureus* and they detected no microbial growth after irradiation [12]. In this study, MW irradiation was used at 650 watts for 3 minutes, and the bristles were not damaged. MW irradiation significantly decreased the number of all tested microorganisms and was the most effective method for* L. rhamnosus*. According to our knowledge,* L. rhamnosus* has not been studied in previous articles about disinfecting toothbrushes.

In recent years, several trademark UV sanitizers have become commercially available. The efficacy of these devices has been studied for bacteria and viruses. Berger et al. used two different UV sanitizers (VIOlight and HIGHDENT) for gram-negative and gram-positive bacteria, and the devices decreased the amount of bacteria by 83% and 100%, respectively [24]. Bélanger-Giguére et al.  reported that the application of the DenTek UV toothbrush sanitizer for 10 minutes was not effective against* S. mutans *[18]. They explained that a longer UV exposure may have eliminated a greater number of microorganisms, but the device they used automatically shut down after 10 minutes. Additionally, the authors emphasized that UV light could not possibly disinfect toothbrushes [18]. In this study, the Dental Total Status Vio Manual sanitizer was used for 20 minutes by manual adjustment according to the manufacturer's recommendations, and the device significantly decreased the number of bacteria. This device was found to be the most effective against* S. mutans* compared to other bacteria. The UV sanitizer used in this study was specific not only for toothbrush disinfection but also for general use. However, the devices used in previous studies were specific for toothbrush disinfection. Differences in the results among the studies may arise from different trademarked products and/or differences between the methods.

Although white vinegar is not commonly used for disinfection in dentistry, this solution is preferred as a promising alternative disinfectant in several areas because of its low toxicity and cost [25]. There are a few studies about the use of white vinegar in dentistry. White vinegar was frequently used in 50% and 100% concentrations to disinfect toothbrushes and acrylic resins [10, 21, 22]. Da Silva et al. reported that 100% white vinegar has good antimicrobial activity against* C. albicans* and* S. aureus* for acrylic resins [21]. This result was supported by Salvia et al., and they remarked that this agent is as effective as 1% NaOCl and 2% chlorhexidine digluconate against* C. albicans*,* E. coli*, and* S. mutans* [22]. In contrast, Komiyama et al. investigated the effectiveness of 50% white vinegar for toothbrush disinfection, and this solution was found to be effective for* S. aureus*,* S. mutans*, and* Streptococcus pyogenes* but not for* C. albicans* [9]. In this study, white vinegar was used in 50% and 100% concentrations for 10 minutes. Both of them were found to be considerably effective for all of the bacteria. Interestingly, 100% white vinegar was the most effective method for* S. mutans* and* S. aureus* and was as effective as 1% NaOCl for* L. rhamnosus *and* E. coli*, which is consistent with the results from Salvia et al. [22].

Propolis is known to be a safe natural bee product and has been used in folk medicine since early times, especially in Europe, due to its antimicrobial, antioxidant, and anti-inflammatory properties [26]. There are many chemical elements, including flavonoids, phenolics, and various aromatic compounds, in propolis. The antioxidant, antibacterial, antifungal, antiviral, and anti-inflammatory features of propolis arise from its flavonoids [26]. Propolis has been reported to be an effective antimicrobial agent against oral pathogens [27]. However, the medical use of propolis is relatively laborious because the concentration of constituents in the solution substantially changes according to the geographic origin, plant sources, proper collection, and handling techniques [28]. The effect of laboratory-manufactured MCP has been investigated for several microorganisms, such as* S. mutans*,* L. rhamnosus*,* C. albicans*, and* Enterococcus faecalis*, in dentistry [11, 28, 29]. Previous studies have shown that MCP is an effective product and is an alternative to chemical mouthwashes for various oral microorganisms due to its nontoxic, natural nature [11, 28, 29]. Additionally, ready-to-use MCP are available on the market. In this study, a ready-to-use mouthwash was used because of its accessibility. There is no study related to these products in the literature, and the MCP used in previous studies were laboratory manufactured. Although a statistically significant difference was found between MCP and the control group, MCP was the least effective agent for all of the microorganisms tested in this study.

## 5. Conclusion

In the present study, different toothbrush disinfection methods were used for* L. rhamnosus*,* S. mutans*,* S. aureus*, and* E. coli*. To date, the influence of toothbrush disinfection methods* on L. rhamnosus* had not been studied. MW was found to be the most effective method for* L. rhamnosus* in this study. There is a need for further studies on* L. rhamnosus* because this microorganism leads to the progression of carious lesions and may also cause bacteremia, meningitis, or endocarditis, particularly in immunocompromised individuals. Additionally, 100% white vinegar was considered to be effective for all of the tested microorganisms and was surprisingly found to be the best method against* S. mutans* and* S. aureus*. White vinegar is nontoxic, cost-effective, easy to access, and appropriate for household use. However, this agent is relatively new in dentistry and may be unknown by many clinicians. Further studies determining all of the effects, including the biocompatibility or toxic effects of vinegar, may increase clinicians' awareness about its antimicrobial capacity, and it might also be introduced to other fields of dentistry, such as root-canal treatment. Similarly, 1% NaOCl is cost-effective and easy to access. Laboratory-manufactured MCP were used in previous studies. There is no study on using MCP for toothbrush disinfection; additionally, ready-to-use MCP has not been investigated in the literature. In this study, MCP was found to be the least effective agent for all of the tested bacteria, although a statistically significant difference was found between MCP and the control group. Therefore, further studies are necessary to determine the efficacy of different trademarked ready-to-use MCP.

## Figures and Tables

**Table 1:** The methods of disinfection examined in this study.

Group	Methods	Time (minutes)
Group 1	Toothbrushes were immersed in 1% NaOCl	10

Group 2	Toothbrushes were placed in MW oven on high power (650 watt)	3

Group 3	Toothbrushes were placed in UV sanitizer on high power	20

Group 4	Toothbrushes were immersed in MCP	10

Group 5	Toothbrushes were immersed in 50% white vinegar	10

Group 6	Toothbrushes were immersed in 100% white vinegar	10

Group 7 (control)	Toothbrushes were washed with tap water	1

**Table 2:** Kruskal Wallis test for *L. rhamnosus*.

Bacteria	Method	Mean	Standard deviation	Minimum	Maximum	Median	Kruskal Wallis test	*P* value	Statistically significant difference
*L. rhamnosus *	1% NaOCl 50% white vinegar 100% white vinegar MW UV MCP Control	3.2 × 10^4^ 5.5 × 10^5^ 1.7 × 10^4^ 2 × 10^3^ 3.4 × 10^6^ 8.2 × 10^6^ 1 × 10^8^	4.6 × 10^4^ 5.7 × 10^5^ 1.9 × 10^4^ 5.2 × 10^3^ 2.2 × 10^4^ 1.9 × 10^6^ 6.8 × 10^7^	10 10 1 1 1.2 × 10^6^ 5.6 × 10^5^ 2.2 × 10^7^	1.2 × 10^5^ 1.2 × 10^6^ 6.4 × 10^4^ 1.7 × 10^4^ 8.8 × 10^6^ 1.2 × 10^7^ 1.9 × 10^8^	4.5 × 10^3^ 4.1 × 10^5^ 1.6 × 10^4^ 5.2 × 10^2^ 2.9 × 10^6^ 8.2 × 10^6^ 9 × 10^7^	5.9 × 10^4^	0.000*	*UV and 1% NaOCl *UV and 50% white vinegar *UV and MW *MCP and 1% NaOCl *MCP and 50% white vinegar *MCP and 100% white vinegar *MCP and MW *MCP and UV *MCP and control *1% NaOCl and control *50% white vinegar and control *100% white vinegar and control *MW and Control *UV and Control

*Statistically significant difference *P* < 0.05.

**Table 3:** Kruskal Wallis test for *S. mutans*.

Bacteria	Method	Mean	Standard deviation	Minimum	Maximum	Median	Kruskal Wallis test	*P* value	Statistically significant difference
*S. mutans *	1% NaOCl 50% white vinegar 100% white vinegar MW UV MCP Control	8 5.6 × 10^2^ 0 3.8 × 10^4^ 4.2 × 10^3^ 1.7 × 10^6^ 4.5 × 10^8^	2.5 × 10 1.3 × 10^2^ 0 1.9 × 10^4^ 8.1 × 10^3^ 2.4 × 10^6^ 1.8 × 10^8^	0 0 0 1.2 × 10^4^ 0 1.1 × 10^4^ 2.2 × 10^8^	8 × 10 4 × 10^2^ 0 7.6 × 10^4^ 2.7 × 10^4^ 7.6 × 10^6^ 7.8 × 10^8^	0 0 0 3.8 × 10^4^ 2.2 × 10^3^ 8.8 × 10^4^ 4.3 × 10^8^	6.1 × 10^4^	0.000*	*MW and 1% NaOCl *MW and 50% white vinegar *MW and 100% white vinegar *MW and UV *MW and control *1% NaOCl and control *50% white vinegar and control *100% white vinegar and control *MW and control *UV and control *MCP and control

*Statistically significant difference *P* < 0.05.

**Table 4:** Kruskal Wallis test for *S. aureus*.

Bacteria	Method	Mean	Standard deviation	Minimum	Maximum	Median	Kruskal Wallis test	*P* value	Statistically significant difference
*S. aureus *	1% NaOCl 50% white vinegar 100% white vinegar MW UV MCP Control	1.4 × 10^2^ 3.3 × 10^4^ 0 1.3 × 10^4^ 1.7 × 10^4^ 1.8 × 10^5^ 4.5 × 10^8^	1.8 × 10^2^ 4.7 × 10^4^ 0 1.9 × 10^3^ 4 × 10^3^ 3.6 × 10^5^ 1.9 × 10^8^	1.2 × 10 1.2 × 10^2^ 0 1.1 × 10^4^ 1.1 × 10^4^ 1.3 × 10^5^ 2.1 × 10^8^	5.1 × 10^2^ 1.2 × 10^5^ 0 1.6 × 10^4^ 2.3 × 10^4^ 2.4 × 10^5^ 8.8 × 10^8^	4.5 × 10 1.8 × 10^3^ 0 1.4 × 10^4^ 1.7 × 10^4^ 1.8 × 10^5^ 4.1 × 10^8^	6.3 × 10^4^	0.000*	*MCP and 1% NaOCl *MCP and 50% white vinegar *MCP and 100% white vinegar *MCP and MW *MCP and UV *Control and 1% NaOCl *Control and 50% white vinegar *Control and 100% white vinegar *Control and MW *Control and UV *Control and MCP

*Statistically significant difference *P* < 0.05.

**Table 5:** Kruskal Wallis test for *E. coli*.

Bacteria	Method	Mean	Standard deviation	Minimum	Maximum	Median	Kruskal Wallis test	*P* value	Statistically significant difference
*E. coli *	1% NaOCl 50% white vinegar 100% white vinegar MW UV MCP Control	5.7 × 10 4.9 × 10^3^ 1.9 × 10^2^ 1.4 × 10^4^ 1.3 × 10^6^ 9.2 × 10^6^ 1 × 10^8^	1.9 × 10 5.5 × 10^3^ 6.7 × 10 2.6 × 10^3^ 1 × 10^5^ 4.4 × 10^6^ 6.7 × 10^7^	2.8 × 10 7.2 × 10 1.1 × 10^2^ 1.1 × 10^4^ 1.7 × 10^6^ 5.9 × 10^6^ 2.2 × 10^7^	8.8 × 10 1.4 × 10^4^ 3.3 × 10^2^ 1.8 × 10^4^ 1.5 × 10^6^ 2.1 × 10^7^ 2 × 10^8^	5.7 × 10 2.1 × 10^3^ 1.8 × 10^2^ 1.4 × 10^4^ 1.3 × 10^6^ 7.8 × 10^6^ 9.3 × 10^7^	6.7 × 10^4^	0.000*	*MCP and 1% NaOCl *MCP and 50% white vinegar *MCP and 100% white vinegar *MCP and MW *MCP and UV *Control and 1% NaOCl *Control and 50% white vinegar *Control and 100% white vinegar *Control and MW *Control and UV *Control and MCP *MW and 1% NaOCl *MW and 50% white vinegar *MW and 100% white vinegar *MW and UV *MW and MCP *UV and 1% NaOCl *UV and 50% white vinegar *UV and 100% white vinegar *UV and MW *UV and MCP

*Statistically significant difference *P* < 0.05.
